# Characterization of gene promoters in pig: conservative elements, regulatory motifs and evolutionary trend

**DOI:** 10.7717/peerj.7204

**Published:** 2019-06-25

**Authors:** Kai Wei, Lei Ma, Tingting Zhang

**Affiliations:** 1 College of Life Science, Shihezi University, Shihezi, Xinjiang, China; 2 Center of Life and Food Sciences Weihenstephan, Technische Universität München, Freising, Byern, Germany

**Keywords:** Sequence conservation, Regulatory motif, Housekeeping promoter, Tissue-specific promoter, Evolutionary dynamics

## Abstract

It is vital to understand the conservation and evolution of gene promoter sequences in order to understand environmental adaptation. The level of promoter conservation varies greatly between housekeeping (HK) and tissue-specific (TS) genes, denoting differences in the strength of the evolutionary constraints. Here, we analyzed promoter conservation and evolution to exploit differential regulation between HK and TS genes. The analysis of conserved elements showed CpG islands, short tandem repeats and G-quadruplex sequences are highly enriched in HK promoters relative to TS promoters. In addition, the type and density of regulatory motifs in TS promoters are much higher than HK promoters, indicating that TS genes show more complex regulatory patterns than HK genes. Moreover, the evolutionary dynamics of promoters showed similar evolutionary trend to coding sequences. HK promoters suffer more stringent selective pressure in the long-term evolutionary process. HK genes tend to show increased upstream sequence conservation due to stringent selection pressures acting on the promoter regions. The specificity of TS gene expression may be due to complex regulatory motifs acting in different tissues or conditions. The results from this study can be used to deepen our understanding of adaptive evolution.

## Introduction

Housekeeping (HK) genes are consistently expressed in different tissues and conditions to maintain basic life activities (Butte, Dzau & Glueck, 2001; Zhu et al., 2008). They may be the minimum collection of genes for normal cellular physiological processes (Kouadjo et al., 2007). Tissue-specific (TS) genes are, in contrast to HK genes, are expressed in specific tissues or conditions and show fluctuant expression levels in different tissues, developmental stages or environments (Kouadjo et al., 2007; Thorrez et al., 2011). Some previous studies reported that significant difference in gene structure, function and evolution between HK and TS genes. For example, HK genes evolve on average more slowly than TS genes (Zhang & Li, 2004), the entropy of TS genes is significantly less than HK genes (Thomas et al., 2015) and the introns, untranslated regions (UTRs) and coding sequences (CDS) of the HK genes are shorter, indicating a selection for compactness in these genes (Eisenberg & Levanon, 2003; Zhu et al., 2008).

The correct performance of function is mainly dependent on complex gene expression regulation which ensure that different genes were expressed in specific tissues, developmental stages and different conditions (Wray et al., 2003). Promoters are the regulatory center in this process, due to a large number of cis-regulatory elements located upstream of a transcription start site (TSS) (Halees, 2003). The key elements related with conservation and gene expression regulation in promoters include short tandem repeat (STR), G-quadruplex sequence (G4), also known as potential quadruplex-forming sequences (PQS) and CpG island, transcription factor binding site, which are often interacted and integrated into combined regulatory motifs to regulate some critical physiological functions (Abe & Gemmell, 2014; Wittkopp & Kalay, 2012; Gemayel et al., 2010). Some studies indicated divergence between promoters of HK and TS genes in structure, conversation and regulation in human and mouse. For example, regulatory motifs of HK and TS promoters showed differently positional bias and conservation in mouse (Bellora, Farré & Albà, 2007; Farré et al., 2007).

In previous studies, empirical results have indicated that nucleotide substitution in regulatory motifs could be one of the causes of phenotypic differentiation (Horton et al., 2014; Andersson, 2009; Xu et al., 2014). The comparisons of upstream promoter sequence across different species have suggested significantly different evolutionary constraints exhibited by promoters of HK and TS genes. In addition, promoters of genes encoding trans-acting factors, such as transcription factors and/or developmental regulatory factors, tend to exhibit especially strong upstream promoter sequence conservation (Lee, Kohane & Kasif, 2005; Iwama & Gojobori, 2004), indicating that the mutations of cis-regulatory elements may change gene expression in different tissues or conditions. Therefore, the evidence of conservation and selection in promoters of different types of gene can contribute to identify HK and TS genes (She et al., 2009). In addition, evolutionary dynamics analysis of promoters can contribute to understanding regulatory patterns and evolutionary trends of HK and TS genes (De Jonge et al., 2007).

The pig (*Sus scrofa*) is an important meat resource and biomedical model. Surveying pig conservation and regulatory patterns in promoters may help pave the way for a greater understanding of the regulatory divergence and evolutionary dynamics in pig HK and TS promoters. Here, we analyzed differences in the conversation of promoters and expression patterns exhibited by HK and TS genes. And the evolutionary dynamics of HK and TS promoters were compared to further understand the reasons for the differences in regulatory patterns. Thus, it is of interest to investigate how evolutionary selection acts on promoters to cause divergent regulation of HK and TS genes.

## Materials and Methods

### Data preparation and definition of HK and TS genes

Gene datasets were defined from pig transcriptome data from 14 RNA-seq projects which includes 21 tissues (heart, spleen, liver, kidney, lung, musculus longissimus dorsi, occipital cortex, hypothalamus, frontal cortex, cerebellum, endometrium, mesenterium, greater omentum, backfat, gonad, ovary, placenta, testis, blood, uterine and lymph nodes) and a total of 131 samples (Table S1). The SRA files of transcriptome data were downloaded from the SRA database of NCBI and then converted to fastq files using fastq-dump in SRA Toolkit (Kodama et al., 2012). Reads of average quality score above 20 were extracted by IlluQC.pl (Patel & Jain, 2012). The filtered reads were mapped to pig reference genome (Sus Sscrofa10.2) using Tophat 2.0.14 (Trapnell, Pachter & Salzberg, 2009). The mapped reads were then submitted to an assembler Cufflinks 2.2.1 to assemble into transcripts and estimate their abundances (Trapnell et al., 2010). The Fragments per Kilobase of exon per Million fragments mapped (FPKM) were calculated to estimate expression level of transcripts.

A total of 3,136 HK genes were defined according to strict criteria (File S1): (i) the transcripts must be detected in all 21 tissues; (ii) the expression variance across tissues were tested by Kolmogorov–Smirnov uniform test, *P* > 0.1 was chosen as the cutoff to extract candidate transcripts; (iii) no abnormal expression in any single tissue; that is, the expression values were restricted within the fourfold range of the average across tissues; and (iv) all transcripts from same candidate gene must met the above criteria. In addition, transcripts with expression restricted to one to three tissues were classified as TS genes, including 1,316 TS genes (File S1). In order to compare the conservative elements and regulatory motifs between HK and TS genes, the two kb upstream sequences of genes were obtained as promoters from Ensemble BioMart (Chen et al., 2010; Kinsella et al., 2011).

### Structure analysis

The structure data of genes, including intron length, 5′ and 3′ UTR length, exon length, CDS length and Transcript length, were obtained from the Ensembl BioMart (Kinsella et al., 2011). The length of various parts between HK and TS genes were compared by Mann–Whitney test (Table 1).

**Table 1:** The structural comparison between HK and TS genes.

Structure	HK gene	TS gene	*P*-value^c^
Total intron length^a^	28,108 ± 173^b^	67,167 ± 691	3.50*E*-182
5′ UTR length	156 ± 3	132 ± 4	2.70*E*-56
3′ UTR length	658 ± 13	499 ± 18	1.30*E*-37
Average exon length per gene	261 ± 3	206 ± 2.63	1.60*E*-19
CDS length	2,181 ± 10	1,475 ± 44	8.40*E*-134
Number of exons	9.2 ± 0.1	15.2 ± 0.68	7.30*E*-61
Transcript length	3,312 ± 13	1,817 ± 40	2.10*E*-79

**Notes:**

a The length was measured in nucleotides.

b The value gives the average and standard error of mean.

c The *P*-value was calculated based on the Mann–Whitney test. UTR, untranslated region; CDS, coding sequence.

### Gene ontology analysis

The functional enrichment of HK and TS genes was performed using DAVID, ver. 6.8 (Huang, Sherman & Lempicki, 2009a, 2009b). All expressed genes in the data were used as background to control accuracy of results. The false discovery rates (FDR) values were calculated to estimate the level of overrepresentation of the selected genes in gene ontology (GO) categories (Storey, 2002). FDR less than 0.01 were used as the cut-off value to acquire significant GO terms.

### Identification of conservative elements

To understand distribution of GC in promoters, we identified CpG islands using the Newcpgreport software (Labarga et al., 2007). The default parameters were chosen to identify CpG islands: (i) the GC content in a 100 bp window exceeded 50%, (ii) the length of CpG island exceeded 200 bp, and (iii) the ratio of observed to expected (O/E) number of CpG islands were must bigger than 0.6 (Gardiner-Garden & Frommer, 1987).

Short tandem repeats were detected in HK and TS promoter sequences using the Phobos 3.3.12 software (Mayer, Leese & Tollrian, 2010). We identified STRs according to following criteria: (i) the STRs identified were must perfect repeats, (ii) repeats units were 2, 3, 4, 5 and 6, (iii) STRs were selected with number of repeat units exceeded six and (iv) the overlapped STRs were counted separately. The mononucleotide repeats were not considered due to repeat number could not be identified.

The Quadruplex forming G-Rich Sequences Mapper was used to detect PQSs in promoters (Kikin, D’Antonio & Bagga, 2006). The search parameters were set as follows: (i) maximum length of PQSs cannot exceed 30, (ii) the minimum number of units in a PQS was four and (iii) the minimum loop size was set as zero. Note that these settings cause some elements to be counted twice in both STRs and PQSs.

### Regulatory motifs discovery by the MEME suite

The protein binding sites and interaction domains are very important features for the regulation of gene expression. The regulatory motifs were found using MEME Suite (Bailey et al., 2009). The following options of input parameters were used: (i) 100 bp bin windows were set to search motifs, (ii) zero or one occurrence per sequence model was chosen to improve the sensitivity and quality of the motif search, (iii) the maximum and minimum width of the motifs were 15 and 6, respectively, (iv) the given promoter sequences or on its reverse complement sequences were searched, (v) the number of motifs was set to five and (vi) 0-order model of sequences was used as the background model (Abe & Gemmell, 2014).

The JASPAR database was used to search biological functions of motifs (Khan et al., 2018).

### Evolutionary features analysis

The evolutionary dynamics of HK and TS CDSs were compared by calculating the substitution ratio. The non-synonymous substitution rate (dN) and synonymous substitution rate (dS) were estimated using the Nei–Gojobori method embedded in MEGA 7.0 (*Z*-test, *P* < 0.05) (Kumar, Stecher & Tamura, 2016; Wei, Zhang & Ma, 2018). The CDSs of HK and TS genes were downloaded from Ensembl BioMart. The orthologous sequences of mouse (*Mus musculus*) were used as outgroups to perform multiple sequence alignments. The following criteria were used: (i) the Overall Average option was chosen, (ii) pairwise deletion was selected to treat Gaps/Missing data. In addition, the orthologous sequences were downloaded from Ensembl BioMart (Kinsella et al., 2011). The dN/dS ratios were calculated to estimate the selective pressure (Hurst, 2002; Dasmeh et al., 2014). In addition, the nucleotide substitution rate of promoters were calculated to estimate conservation of promoters.

Statistical analyses involved in present study were performed in R (www.r-project.org).

## Results

### Identification of HK and TS genes

In our previous study, 3,136 genes were defined as HK genes, which maintain relatively stable expression level in all 21 tissues (File S1; Wei, Zhang & Ma, 2018). The 1,316 genes defined as TS genes contained 2,214 transcripts expressing in one to three tissues (File S1).

**Figure 1:**
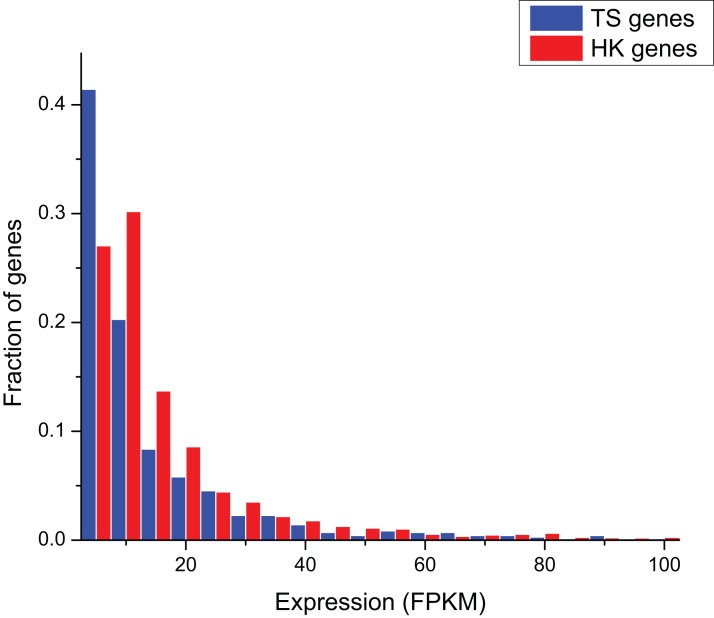
The comparison of expression level between HK and TS genes.

The comparison of gene expression in ERP002055 sequencing project indicates that the average expression level of HK genes (FPKM = 17.10 ± 3.63) was significantly higher than TS genes (FPKM = 6.43 ± 64.08) (Mann–Whitney test, *P* < 0.01) (Fig. 1).

### The structural and functional comparison of HK and TS genes

There are significant differences between HK and TS in gene structural length (Mann–Whitney test, *P* < 0.01, Table 1). The total length and intron length of TS genes are significantly longer than HK genes, but other structures are significantly shorter than HK genes, such as UTR and CDS. These results indicated that the structure of HK genes is more compact than TS genes. Combined with expression level analysis, the high expression characteristics of HK genes may require a flexible gene structure, that is, a more compact gene structure enables it to initiate expression quickly, and it takes less time and energy in the expression process.

In addition, TS genes displayed a higher number of exons and transcripts compared with HK genes (Mann–Whitney test, *P* < 0.01), which may be related to the spatiotemporal dependence of TS genes that express different splicing isoforms at different developmental stages of the cell or in different environmental conditions.

The GO enrichment analysis of biological processes revealed that the functions of HK genes are mainly concentrated on the basal metabolism of cells, such as energy metabolism, cellular transport and synthesis and decomposition of macromolecules (Table S2). The principal functions of TS genes are related to tissue specificity, such as many genes enriched to tissue differentiation and development, and many genes are associated with cellular immune response (Table S3). The results showed that HK genes and TS genes have their own specific functional characteristics, and their roles in cells are significantly different. TS genes are genes that distinguish between tissues. HK genes mainly provides the necessary substances and energy in the cells to perform basic life activities. HK and TS genes gradually form unique functional characteristics in the long-term evolutionary process, and their mutual cooperation is the basis for the orderly operation of cell life activities.

### GC content and CpG island density in HK and TS promoters

**Figure 2:**
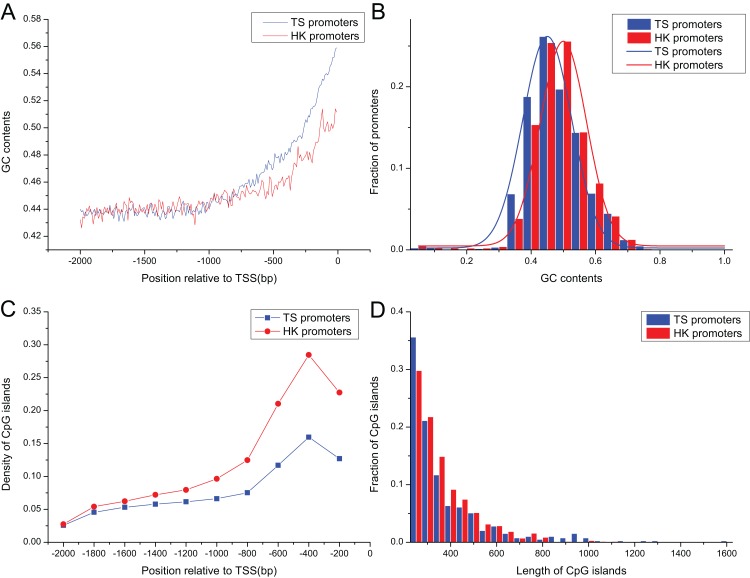
The distribution of content and length of GC and CpG islands between HK and TS promoters. (A) The tendency of GC contents in the promoters (the mean and standard error are 0.46 ± 0.0015 and 0.45 ± 0.0024 in HK and TS promoters, respectively), (B) the distribution of GC contents, (C) the distribution of CpG islands (the averaged density in HK and TS promoters are 0.47 ± 0.0013 and 0.30 ± 0.0016, respectively), (D) the distribution of length of CpG islands (the averaged length in HK and TS promoters are 352 ± 9.82 and 234 ± 3.36, respectively).

Promoter sequences of pig HK and TS genes increased gradually as it approached the TSS in their GC contents (Fig. 2A), ranging from 0.30 to 0.75, and their averages were 0.46 and 0.45, respectively. GC contents in HK promoters were significantly higher than TS promoters as it approached the TSS (Mann–Whitney test, *P* < 0.01) (Fig. 2B).

The 1,556 CpG islands were identified in HK promoters with a density of 0.47 per promoter. TS promoters contained 393 CpG islands with a density of 0.30. Figure 2C shows that the density of CpG islands in HK promoters is higher than TS promoters (Mann–Whitney test, *P* < 0.01). In addition, the analysis showed that the length of CpG islands in HK promoters is longer than TS promoters (Mann–Whitney test, *P* < 0.01) (Fig. 2D). The higher GC and CpG island content may indicate HK promoters are more stable than the TS promoters. HK genes with high density CpG islands have higher transcriptional activity across tissues, that is, it has a higher level of expression, while TS genes may be restricted by strict expression in specific tissues (Fenouil et al., 2012; Vavouri & Lehner, 2012).

### Abundance of STR and PQS in HK and TS promoters

**Figure 3:**
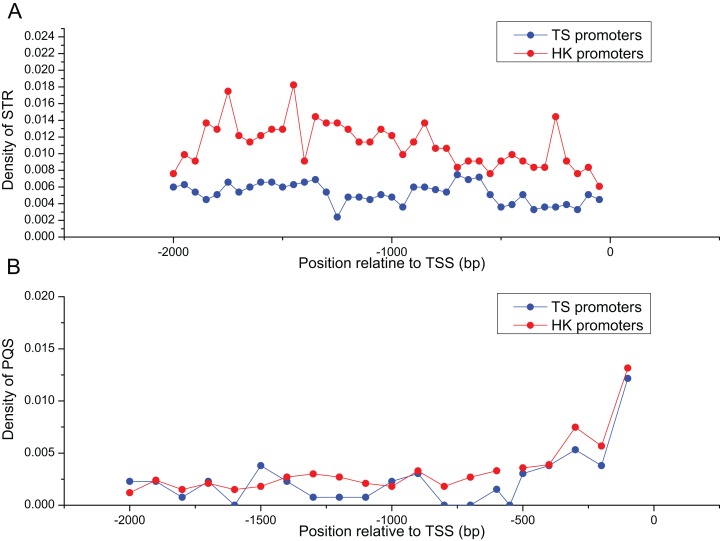
The distribution of STR and PQS between HK and TS promoters. (A) The distribution of STR density in the promoters, (B) the distribution of PQS density in the promoters (the mean and standard error are 0.0028 ± 0.00061 and 0.0024 ± 0.00058).

Table 2 summarized the frequencies of STR motifs in HK and TS promoters. The similar STR motifs were detected in HK and TS promoters. However, STR motifs density in HK promoters was significantly higher than TS promoters (Table 2, Mann–Whitney test, *P* < 0.01). Figure 3A indicated STR density of HK promoters significantly higher than TS promoters (Mann–Whitney test, *P* < 0.01). In addition, the distribution of PQS between HK and TS promoters were no significant difference. But PQS content in the proximal part of promoter was higher than the distal part of the promoter (Fig. 3B).

**Table 2:** The comparison of STR between HK and TS promoters.

STR	TS promoters		HK promoters	
	Number of STR	Frequency of STR	Number of STR	Frequency of STR
AC	76	0.058	393	0.13
AG	30	0.023	160	0.051
AT	34	0.026	145	0.046
CG	5	0.0035	10	0.0030
AAC	20	0.015	74	0.024
AAG	1	0.00064	14	0.0046
AAT	4	0.0029	24	0.0076
ACC	0	0	7	0.0023
AGG	3	0.0026	5	0.0015
AGC	2	0.0013	10	0.0030
CCG	12	0.0089	12	0.0038
ACAG	0	0.00032	5	0.0015
AAGG	0	0.00032	19	0.0061
AATC	1	0.00064	0	0
AAAC	3	0.0022	14	0.0046
AAAG	2	0.0016	31	0.0099
AAAT	8	0.0061	24	0.0076
AGAT	1	0.00096	2	0.00076
AGGG	1	0.00064	5	0.0015
ATCC	0	0	7	0.0023
AAAAG	1	0.00032	5	0.0015
AAAAT	1	0.00032	5	0.0015
STR/seq^a^		0.15		0.31

**Note:**

a STR/seq is the number of STR motif counted per promoter sequence.

### Regulatory motifs identified in the HK and TS promoters

Motif density and types of TS promoters were significantly higher than HK promoters (Mann–Whitney test, *P* < 0.01). A total of 38 types of regulatory motifs were identified in HK promoters, a total of 74,322, with a density of 23 motifs per promoter (Table 3; Table S4). There were 115 types of regulatory motifs in the TS promoters, a total of 67,123, with a density of 51 motifs per promoter (Table 4; Table S5). These results are consistent with variable expression levels and patterns of TS genes in different tissues and conditions. In HK and TS promoters, some motifs are zinc finger factors, especially in HK promoters. The functions of HK motifs are partially similar with TS motifs, for examples some C2H2 zinc finger factors but different motifs are chosen to bind the same transcription factor.

**Table 3:** The top 10 of regulatory motifs in HK promoters.

Motif	Length	Number of motifs	*E*-value	Description
GCYRCAGC	8	3,637	3.8*E*-403	C2H2 zinc finger factors
GCCHGGGA	8	2,991	1.8*E*-353	Rel homology region (RHR) factors
GCTGTRGC	8	2,256	1.4*E*-313	C2H2 zinc finger factors
TCCCWGGC	8	2,521	1.1*E*-298	Rel homology region (RHR) factors
TCSTTAAC	8	1,896	1.7*E*-263	Tryptophan cluster factors
GGAACTYC	8	1,882	1.4*E*-242	Rel homology region (RHR) factors
AAAAWAAA	8	4,404	3.4*E*-229	C2H2 zinc finger factors
GTGGTGTA	8	1,282	9*E*-228	C4 zinc finger factors
TACACCAC	8	1,310	8.4*E*-226	C4 zinc finger factors
CATATGS	7	2,634	4.7*E*-224	Basic helix-loop-helix factors

**Note:**

The top10 regulatory motifs in HK promoters were listed in table. N or X: A G C T; V: A C T; H: A C T; D: A G T; B: C G T; M: A C; R: A G; W: A T; S: C G; Y: C T; K: G T.

**Table 4:** The top 10 of regulatory motifs in TS promoters.

Motifs	Length	Number of motifs	*E*-value	Description
GCYACAGC	8	806	2.40*E*-112	C2H2 zinc finger factors
GCCHGGGA	8	948	3.50*E*-99	Fork head/winged helix factors
GCTGTRGC	8	703	1.10*E*-97	C2H2 zinc finger factors
AAAAWAAA	8	1,668	1.80*E*-92	C2H2 zinc finger factors
TATWTAT	7	1,055	8.80*E*-85	MADS box factors
TCSTTAAC	8	584	7.90*E*-77	Tryptophan cluster factors
TACACCAC	8	413	6.60*E*-71	C4 zinc finger factors
TTTTTYTT	8	1,630	9.80*E*-74	C2H2 zinc finger factors
CATATGS	7	896	1.60*E*-67	Basic helix-loop-helix factors
CCACTGAG	8	517	1.90*E*-63	Nuclear receptors with C4 zinc

**Note:**

The top10 regulatory motifs in TS promoters were listed in table. N or X: A G C T; V: A C T; H: A C T; D: A G T; B: C G T; M: A C; R: A G; W: A T; S: C G; Y: C T; K: G T.

In addition, there are 22 and 99 specific regulatory motifs in HK and TS promoters, respectively. But only 16 types of regulatory motifs were shared between them. These results indicated a large number of specific regulatory motifs in TS promoters which may help TS genes to adapt to different conditions.

### Divergence of HK and TS promoter sequences

The promoters of genes show sequence divergence (Lee, Kohane & Kasif, 2005; Iwama & Gojobori, 2004; Suzuki et al., 2004). The level of promoter sequence divergence is positively correlated with the evolutionary rate of the encoded protein (Castillo-Davis, Hartl & Achaz, 2004; Chin, Chuang & Li, 2005). To investigate evolutionary dynamic of HK and TS promoters, the number of non-synonymous substitutions per non-synonymous site (dN), the number of synonymous substitutions per synonymous site (dS) and dN/dS ratio were calculated for HK and TS CDS using mouse (*Mus musculus*) as an outgroup. And the promoter nucleotide substitution rate (dP) was also estimated to understand the evolutionary trend of promoters in pig (Files S2 and S3).

**Figure 4:**
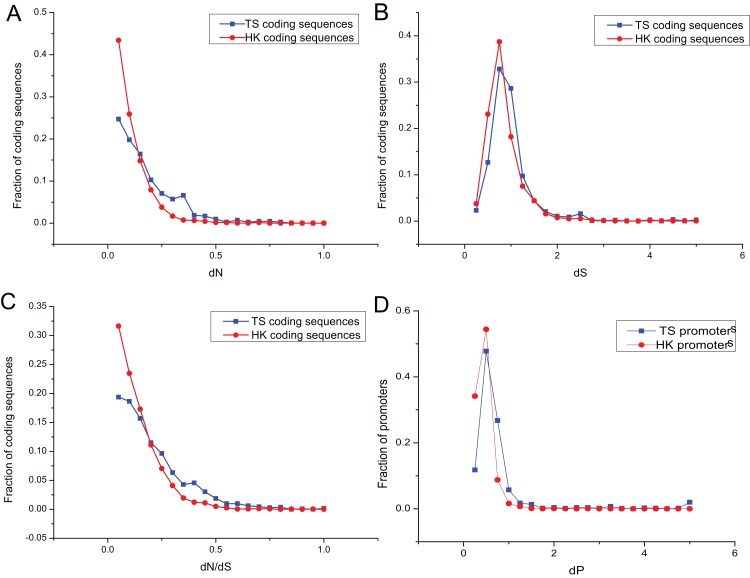
The overlap of regulatory motifs between HK and TS promoters. Evolutionary dynamics of promoters and coding sequence. (A–C) The distribution of dN, dS and dN/dS in coding sequences, (D) the distribution of dP in promoters.

Evolutionary dynamic analysis showed that the vast majority dN and dN/dS of CDS, were less than one, showing a power-law distribution, indicating that most of the CDS were under the purifying selection pressure and in negative selection (Figs. 4A and 4C; Table S6). The dS showed an approximately normal distribution and was significantly greater than dN (Mann–Whitney test, *P* < 0.01). About 20% of CDS had dS greater than one (Fig. 4B). In addition, dP of TS promoters (0.64) was significantly higher relative to that of HK promoters (Fig. 4D; Table S6), which indicated HK promoters with increased conservation and suffered more stringent selection pressure than TS promoters.

Interestingly, the nucleotide substitution rate of promoters showed significantly positive correlation with the CDS (for HK genes, dP and dN, *r* = 0.23, *P* < 10^−32^; dP and dN/dS, *r* = 0.16, *P* < 10^−38^; dP and dS, *r* = 0.38, *P* < 10^−37^; and for TS genes dP and dN, *r* = 0.27, *P* < 10^−36^; dP and dN/dS, *r* = 0.23, *P* < 10^−32^; dP and dS, *r* = 0.44, *P* < 10^−41^). Therefore, promoters showed a similar tendency with the CDS.

The nucleotide substitution rate of HK promoters was significantly smaller than that of TS promoters. The structure of HK promoters became more stable and evolved slower than TS promoters, which were determined by the importance of HK genes in cells (Nei & Kumar, 2000). The evolution of TS promoters is significantly faster than that of HK promoters, indicating weaker selection pressures can help specific tissues adapt to different environmental conditions (Zhang & Li, 2004).

## Discussion

The present study characterized conservative motifs and regulatory elements of gene promoters in pig. In addition, combined with the analysis of evolutionary dynamics, we investigated the difference of HK and TS genes in regulation of gene expression.

In the long-term evolution and environmental adaptation process, HK and TS genes gradually form specific genomic structure, respectively. HK genes showed more compact structures than TS genes. This may be due to different properties of gene expression (Chang et al., 2011; Hong et al., 2013). TS genes showed shorter transcript length, but a higher number of transcripts and exons indicated that more alternative splicing occurs in expression to adapt to different environments. This may contribute to the expression of HK genes activated at any time to maintain the basic life activities of the cells (Eisenberg & Levanon, 2003, 2013). For example, genes associated with ribosome complex are continuously expressed in the cells to meet the requirements of the body protein (Brandman et al., 2012). However, TS genes only express at specific developmental stages of a particular tissue, and their ultimate goal is to adapt to temporal and spatial development of tissues (Holder & Klein, 1999; Lawson & Zhang, 2008). For example, EPHB3 (EPH receptor B3) gene is expressed in the nervous system, which is mainly involved in the development of neurons (Holder & Klein, 1999).

In the process of evolution, the HK promoters are under strict purifying selection pressure, and the gene expression level tends to be stable in different tissues and environments to maintain life, while constrained forces of TS promoters in evolution is much smaller than HK promoters. In addition, nucleotide substitution rate of TS promoters is significantly higher than HK promoters. The adaptability is mainly reflected in the phenotypic changes, so the adaptability of the organism is mainly reflected in the selective expression of TS genes under different environmental conditions (Hill et al., 1998). This also explains the reason that the higher nucleotide substitution of TS promoters. The evolution of TS promoters and selective expression are the embodiment of environmental adaptability, while the evolution of HK promoters and the stability of expression aim to maintain the basic cellular function and in different tissues and conditions (Urrutia & Hurst, 2001).

The regulatory elements on promoter are important factors which can contribute to species adaptation to changing environments. The HK promoters of pig shows higher sequence conservation than TS promoters, mainly due to the strict purifying selection pressure act on HK promoters to maintain the stability of HK gene expression in different environments. The expression of TS genes is selective, and it is selectively expressed and fluctuating under different conditions, which requires the promoter to initiate different regulatory pathways under different conditions. So the expression of genes can be regulated at any time to adapt to the current environment (Larsen et al., 2013; Urrutia & Hurst, 2001).

The conserved sequences (STR, PQS and CpG island) in the HK promoters are higher than TS promoters. Genes driven and regulated by repeat sequence promoters are indicated to show significantly higher rates of transcription than those without repeat elements as reported by experiments showing that knockout of STR elements in promoters show significant differences in gene expression compared with promoters without having knocked out STR (Vinces et al., 2009; Valipour et al., 2013). Promoters with CpG islands show high transcriptional activity in multiple tissues (Elango & Yi, 2011; Sharif et al., 2010). The relationship between gene ontologies and CpG islands length suggest the important role of CpG islands in chromatin structures by methylation (Robertson, 2002). The regulation of HK genes is relatively simple compared to TS gene regulation because HK genes are continuously expressed under any conditions (Bao, Li & Zhao, 2012; Bellora, Farré & Albà, 2007). TS genes are differentially expressed at different developmental stages and conditions, and are effector genes that adapt to different environments. They have different isoforms and expression levels under different conditions and need a large number of different regulatory motifs to bind different transcription factors to regulate gene expression (Murakami, Kojima & Sakaki, 2003). For example, the UCL1 (Urothelial cancer associated 1 conserved region) gene, which is specifically expressed in the bladder, is regulated under normal conditions by the transcription factor C/EBPα binding to the promoter, but transcription factor HIF-1α (Hypoxia-inducible factor 1 alpha) plays a major role in the regulation of UCA1 gene expression under conditions of cellular hypoxia (Wang et al., 2006, 2008).

## Conclusions

In the long-term evolution process, HK genes and TS genes showed significant differences in evolutionary constraint and evolutionary trend. HK promoters are more conservative than TS promoters. TS genes exhibited more complex regulatory patterns than HK genes. The adaptation of organisms to different environments may be achieved through the regulation of genes by TS motifs.

## Supplemental Information

10.7717/peerj.7204/supp-1 Supplemental Information 1 The summary of the raw RNA-Seq data used in this study from the SRA database, including accession numbers and tissues.

10.7717/peerj.7204/supp-2 Supplemental Information 2 The enrichment of biological process in HK genes. ^a^*P*_FDR_ were corrected by false discovery rates base on the fisher’s exact test *P*-value.

10.7717/peerj.7204/supp-3 Supplemental Information 3 The enrichment of biological process in TS genes. ^a^*P*_FDR_ were corrected by false discovery rates base on the fisher’s exact test *P*-value.

10.7717/peerj.7204/supp-4 Supplemental Information 4 The detection of regulatory motif in HK promoters. Note: The regulatory motifs in HK promoters were listed in table. N or X: A G C T; V: A C T; H: A C T; D: A G T; B: C G T; M: A C; R: A G; W: A T; S: C G; Y: C T; K:G T.

10.7717/peerj.7204/supp-5 Supplemental Information 5 The detection of regulatory motif in TS promoters. Note: The regulatory motifs in TS promoters were listed in table. N or X: A G C T; V: A C T; H: A C T; D: A G T; B: C G T; M: A C; R: A G; W: A T; S: C G; Y: C T; K:G T.

10.7717/peerj.7204/supp-6 Supplemental Information 6 Evolutionary features of coding sequences and promoters. ^a^The value gives the average and standard error of mean. ^b^The *P*-value was calculated based on the Mann-Whitney test.

10.7717/peerj.7204/supp-7 Supplemental Information 7 HK and TS genes. Lists of all paired HK and TS genes.

10.7717/peerj.7204/supp-8 Supplemental Information 8 Substitution rates of HK coding sequences and promoters. Lists of dN, dS, dN/dS and dP in HK coding sequences and promoters.

10.7717/peerj.7204/supp-9 Supplemental Information 9 Substitution rates of TS coding sequences and promoters. Lists of dN, dS, dN/dS and dP in TS coding sequences and promoters.

## Additional Information and Declarations

### Data Availability

The following information was supplied regarding data availability: The details of the third-party datasets are available in Table S1.
