# Supplementary material for: Characterization of gene promoters in pig: conservative elements, regulatory motifs and evolutionary trend
Source: PeerJ. 2019 Jun 25;7:e7204. doi: 10.7717/peerj.7204 (PMC6598670; doi:10.7717/peerj.7204)
Supplement: Supplemental Information 1 [file peerj-07-7204-s001.docx]

| SRA accession | Number of samples | Number of tissues | Number of reads(million) | Name of tissues |
| --- | --- | --- | --- | --- |
| SRP067181 | 10 | 1 | 877 | liver |
| SRP018856 | 3 | 3 | 263 | Backfat, greater omentum, mesenterium |
| SRP008516 | 2 | 1 | 50 | gonad |
| SRP032451 | 10 | 1 | 839 | hypothalamus |
| SRP026594 | 6 | 2 | 34 | Backfat, musculus longissimus dorsi |
| SRP018288 | 4 | 4 | 190 | heart, liver, lung, kidney |
| SRP018102 | 8 | 1 | 247 | endometrium |
| SRP058401 | 6 | 1 | 366 | ovary |
| SRP014902 | 3 | 3 | 59 | placenta, testis, Pool of 11 tissues |
| ERP013761 | 31 | 1 | 632 | blood |
| SRP062860 | 12 | 1 | 797 | uterine |
| SRP065485 | 8 | 1 | 91 | lymph nodes |
| SRP027378 | 8 | 1 | 272 | endometrium |
| ERP002055 | 20 | 10 | 223 | heart, spleen, liver, kidney, lung, musculus longissimus dorsi, occipital cortex, hypothalamus, frontal cortex, cerebellum |
