# Supplementary material for: Characterization of gene promoters in pig: conservative elements, regulatory motifs and evolutionary trend
Source: PeerJ. 2019 Jun 25;7:e7204. doi: 10.7717/peerj.7204 (PMC6598670; doi:10.7717/peerj.7204)
Supplement: Supplemental Information 2 — aPFDR were corrected by false discovery rates base on the fisher’s exact test P-value. [file peerj-07-7204-s002.docx]

| GO term ID | Biological process | Number of genes | Frequency of genes（%） | *P*_FDR_ |
| --- | --- | --- | --- | --- |
| 0044260 | cellular macromolecule metabolic process | 998 | 39.04 | 1.06E-69 |
| 0043170 | macromolecule metabolic process | 1037 | 40.57 | 1.56E-64 |
| 0034641 | cellular nitrogen compound metabolic process | 785 | 30.71 | 1.76E-52 |
| :0006139 | nucleobase-containing compound metabolic process | 686 | 26.83 | 3.58E-45 |
| 0046483 | heterocycle metabolic process | 695 | 27.19 | 5.25E-45 |
| 0006725 | cellular aromatic compound metabolic process | 695 | 27.19 | 4.49E-43 |
| 1901360 | organic cyclic compound metabolic process | 698 | 27.31 | 6.37E-40 |
| 0022613 | ribonucleoprotein complex biogenesis | 123 | 4.81 | 3.54E-29 |
| 0019538 | protein metabolic process | 562 | 21.98 | 7.71E-20 |
| 0044249 | cellular biosynthetic process | 600 | 23.47 | 1.52E-16 |
| 0006996 | organelle organization | 432 | 16.90 | 3.98E-16 |
| 1901576 | organic substance biosynthetic process | 601 | 23.51 | 4.72E-15 |
| 0046907 | intracellular transport | 185 | 7.23 | 7.62E-12 |
| 0051171 | regulation of nitrogen compound metabolic process | 400 | 15.64 | 1.01E-10 |
| 0043933 | macromolecular complex subunit organization | 245 | 9.58 | 1.31E-09 |
| 0070727 | cellular macromolecule localization | 197 | 7.70 | 1.48E-09 |
| 0033554 | cellular response to stress | 208 | 8.13 | 5.39E-09 |
| 0006403 | RNA localization | 37 | 1.44 | 6.40E-09 |
| 0051649 | establishment of localization in cell | 206 | 8.05 | 3.64E-08 |
| 0009889 | regulation of biosynthetic process | 375 | 14.67 | 1.30E-07 |
