# Supplementary material for: Characterization of gene promoters in pig: conservative elements, regulatory motifs and evolutionary trend
Source: PeerJ. 2019 Jun 25;7:e7204. doi: 10.7717/peerj.7204 (PMC6598670; doi:10.7717/peerj.7204)
Supplement: Supplemental Information 3 — aPFDR were corrected by false discovery rates base on the fisher’s exact test P-value. [file peerj-07-7204-s003.docx]

| GO term ID | Biological process | Number of genes | Frequency of genes（%） | *P*_FDR_ |
| --- | --- | --- | --- | --- |
| 0050877 | neurological system process | 65 | 7.88 | 4.10E-52 |
| 0070489 | T cell aggregation | 26 | 3.15 | 7.54E-34 |
| 0007154 | cell communication | 226 | 27.42 | 2.74E-21 |
| 0046649 | lymphocyte activation | 31 | 3.76 | 1.03E-16 |
| 0070661 | leukocyte proliferation | 19 | 2.30 | 3.62E-16 |
| 0050906 | detection of stimulus involved in sensory perception | 24 | 2.91 | 3.47E-11 |
| 1903708 | positive regulation of hemopoiesis | 10 | 1.21 | 1.56E-09 |
| 0060322 | head development | 25 | 3.03 | 3.93E-09 |
| 0051094 | positive regulation of developmental process | 39 | 4.73 | 4.75E-08 |
| 0007517 | muscle development | 53 | 6.43 | 9.42E-08 |
| 0050776 | regulation of immune response | 23 | 2.791 | 3.77E-07 |
| 0048732 | gland development | 17 | 2.06 | 3.93E-06 |
| 0007420 | brain development | 23 | 2.79 | 5.05E-06 |
| 0030324 | lung development | 46 | 5.58 | 5.90E-06 |
| 0007275 | multicellular organism development | 134 | 16.26 | 1.30E-05 |
| 0019953 | sexual reproduction | 24 | 2.91 | 2.92E-05 |
| 0051962 | positive regulation of nervous system development | 16 | 1.94 | 3.13E-05 |
| 0046883 | regulation of hormone secretion | 10 | 1.2 | 3.20E-05 |
| 0001889 | liver development | 29 | 3.51 | 6.68E-05 |
| 0007507 | heart development | 17 | 2.06 | 1.26E-04 |
