# Supplementary material for: Characterization of gene promoters in pig: conservative elements, regulatory motifs and evolutionary trend
Source: PeerJ. 2019 Jun 25;7:e7204. doi: 10.7717/peerj.7204 (PMC6598670; doi:10.7717/peerj.7204)
Supplement: Supplemental Information 4 — Note: The regulatory motifs in HK promoters were listed in table. N or X: A G C T; V: A C T; H: A C T; D: A G T; B: C G T; M: A C; R: A G; W: A T; S: C G; Y: C T; K:G T. [file peerj-07-7204-s004.docx]

| motifs | Length of motifs | Number of motifs | E value |
| --- | --- | --- | --- |
| GCYRCAGC | 8 | 3637 | 3.8e-403 |
| GCCHGGGA | 8 | 2991 | 1.8e-353 |
| GCTGTRGC | 8 | 2256 | 1.4e-313 |
| TCCCWGGC | 8 | 2521 | 1.1E-298 |
| TCSTTAAC | 8 | 1896 | 1.7E-263 |
| GGAACTYC | 8 | 1882 | 1.4E-242 |
| AAAAWAAA | 8 | 4404 | 3.4E-229 |
| GTGGTGTA | 8 | 1282 | 9E-228 |
| TACACCAC | 8 | 1310 | 8.4E-226 |
| CATATGS | 7 | 2634 | 4.7E-224 |
| TTTWTTTT | 8 | 4945 | 5.8E-217 |
| CACTGMGC | 8 | 1713 | 2E-211 |
| GTGGGTTA | 8 | 1214 | 7.6E-206 |
| CCTCATGG | 8 | 1180 | 9.8E-193 |
| GGATCYGR | 8 | 1803 | 4.5E-188 |
| GGAGTTCC | 8 | 1174 | 1.9E-175 |
| CCATGAGG | 8 | 944 | 2.7E-139 |
| CTAGTCRG | 8 | 921 | 2.1E-134 |
| TAGGGGTC | 8 | 809 | 1E-121 |
| CACRGCAA | 8 | 1041 | 6.9E-118 |
| GAATCNGA | 8 | 1265 | 5.5E-116 |
| GACCCCTA | 8 | 767 | 7.2E-113 |
| GCKCAGTG | 8 | 895 | 7.5E-109 |
| CCSCDCCC | 8 | 2888 | 2.1E-110 |
| ATAWATA | 7 | 2308 | 6.6E-101 |
| TCTGYRAC | 8 | 1312 | 2.1E-122 |
| GGGHGGGG | 8 | 2196 | 2.8E-93 |
| TTTWAAAA | 8 | 1823 | 5.3E-92 |
| AARASAAA | 8 | 2897 | 6.7E-87 |
| YCRGATCC | 8 | 1240 | 1.2E-95 |
| CTGHGCCA | 8 | 1190 | 3.1E-85 |
| TGCYGTGA | 8 | 811 | 1.7E-81 |
| CGCSGS | 6 | 5098 | 9E-79 |
| TKTSTTTT | 8 | 3291 | 2.6E-77 |
| GTCRCAGA | 8 | 641 | 1.8E-76 |
| AACCCRCA | 8 | 790 | 1.1E-72 |
| GGYGTAGG | 8 | 551 | 1.6E-69 |
| TATWTWT | 7 | 3802 | 2.9E-67 |
| Number of total motifs |  | 74322 |  |
| Number of motifs per promoter |  | 23 |  |
