# Supplementary material for: Characterization of gene promoters in pig: conservative elements, regulatory motifs and evolutionary trend
Source: PeerJ. 2019 Jun 25;7:e7204. doi: 10.7717/peerj.7204 (PMC6598670; doi:10.7717/peerj.7204)
Supplement: Supplemental Information 5 — Note: The regulatory motifs in TS promoters were listed in table. N or X: A G C T; V: A C T; H: A C T; D: A G T; B: C G T; M: A C; R: A G; W: A T; S: C G; Y: C T; K:G T. [file peerj-07-7204-s005.docx]

| Motifs | Length of motifs | Number of motifs | E value |
| --- | --- | --- | --- |
| GCYACAGC | 8 | 806 | 2.40E-112 |
| GCCHGGGA | 8 | 948 | 3.50E-99 |
| GCTGTRGC | 8 | 703 | 1.10E-97 |
| AAAAWAAA | 8 | 1668 | 1.80E-92 |
| TCCCDGGC | 8 | 875 | 8.80E-85 |
| TCSTTAAC | 8 | 584 | 7.90E-77 |
| TACACCAC | 8 | 413 | 6.60E-71 |
| TTTTTYTT | 8 | 1630 | 9.80E-74 |
| CATATGS | 7 | 896 | 1.60E-67 |
| CCACTGAG | 8 | 517 | 1.90E-63 |
| GGAACTCC | 8 | 457 | 6.90E-63 |
| GTGGTRTA | 8 | 446 | 8.50E-56 |
| GTGGGTTA | 8 | 343 | 2.10E-52 |
| GGAGTTCC | 8 | 355 | 1.00E-44 |
| TAGGGGTC | 8 | 276 | 1.20E-41 |
| TATWTAT | 7 | 1055 | 1.70E-41 |
| CCCCDCCC | 8 | 829 | 9.60E-40 |
| CTCATGGW | 8 | 448 | 1.70E-37 |
| GACCCCTA | 8 | 223 | 2.50E-37 |
| GGGYGGGG | 8 | 577 | 3.20E-37 |
| ATCBGAGC | 8 | 378 | 1.90E-36 |
| TGTSTGTG | 8 | 508 | 3.90E-36 |
| TTTWWAAA | 8 | 1055 | 4.10E-36 |
| GATCYGGC | 8 | 300 | 1.80E-31 |
| CCATGASG | 8 | 331 | 4.90E-31 |
| CACRGCAA | 8 | 358 | 7.60E-31 |
| CTAGTCRG | 8 | 269 | 1.30E-30 |
| GCDGCWGC | 8 | 701 | 4.00E-30 |
| ACMCACA | 7 | 854 | 1.50E-29 |
| GAATCNGA | 8 | 377 | 7.80E-30 |
| AARASAAA | 8 | 1134 | 8.20E-28 |
| TGHGCCAC | 8 | 357 | 2.50E-27 |
| GATCCHGC | 8 | 303 | 5.20E-25 |
| TAWATA | 6 | 1756 | 6.50E-25 |
| YGYGGCTC | 8 | 371 | 3.30E-27 |
| TATTTBY | 7 | 2483 | 5.00E-25 |
| TCTGYRAC | 8 | 389 | 8.60E-26 |
| GTYRCAGA | 8 | 392 | 1.60E-26 |
| CCRTGAGC | 8 | 229 | 1.40E-22 |
| CGAACCYG | 8 | 159 | 3.20E-20 |
| CGCGSG | 6 | 634 | 6.00E-19 |
| CTCTSTS | 7 | 1419 | 5.90E-21 |
| TTBTCTTT | 8 | 690 | 1.00E-18 |
| GGCCRCAC | 8 | 195 | 4.40E-18 |
| GCTGYG | 6 | 1391 | 4.90E-17 |
| GGGSMGGG | 8 | 707 | 1.80E-17 |
| GAGRARGA | 8 | 564 | 5.50E-17 |
| GTGGTTAA | 8 | 160 | 4.70E-16 |
| GCGTAGGC | 8 | 82 | 5.90E-15 |
| STGTGGCC | 8 | 215 | 2.10E-14 |
| GCCGCKCC | 8 | 149 | 6.80E-14 |
| CRCAGC | 6 | 1280 | 9.00E-14 |
| AAWAAWA | 7 | 1549 | 2.30E-15 |
| ACGCCRGA | 8 | 125 | 2.40E-12 |
| TAHATGTA | 8 | 236 | 1.70E-13 |
| CGYGG | 5 | 1816 | 1.70E-11 |
| ASAGASA | 7 | 1216 | 1.70E-13 |
| TTTTRTTT | 8 | 504 | 2.60E-11 |
| RGAAATR | 7 | 1314 | 9.50E-13 |
| YGGGTTCG | 8 | 106 | 2.30E-10 |
| CSGCCKCC | 8 | 258 | 1.40E-12 |
| CTYGCTCA | 8 | 265 | 2.60E-10 |
| KAAWATA | 7 | 983 | 3.50E-10 |
| RCTGTACA | 8 | 154 | 8.60E-10 |
| TCYGATTC | 8 | 197 | 2.30E-09 |
| GTGCMGCC | 8 | 135 | 2.70E-09 |
| TTTCTCY | 7 | 833 | 3.00E-09 |
| GGCGGCGG | 8 | 98 | 1.20E-08 |
| TYCCTKCC | 8 | 537 | 2.90E-10 |
| GTRTSTG | 7 | 682 | 2.70E-08 |
| TTTCCTBT | 8 | 464 | 4.30E-08 |
| ATACABAT | 8 | 168 | 6.80E-08 |
| CTGTGNC | 7 | 592 | 1.00E-07 |
| WATATTT | 7 | 416 | 2.90E-07 |
| TCYGGTGT | 8 | 172 | 7.00E-07 |
| GCTGCACC | 8 | 130 | 2.30E-06 |
| CCAGCCTM | 8 | 236 | 3.30E-06 |
| AACGCWGG | 8 | 95 | 4.30E-06 |
| TATAMA | 6 | 1157 | 7.80E-06 |
| GAGVGAGG | 8 | 337 | 1.00E-05 |
| TATWTTA | 7 | 519 | 1.50E-05 |
| CTRGGAA | 7 | 715 | 2.70E-05 |
| GSAGGTG | 7 | 611 | 4.00E-05 |
| CTBCCTC | 7 | 1055 | 4.10E-05 |
| WTTTTA | 6 | 2418 | 1.70E-05 |
| CACGT | 5 | 778 | 5.90E-05 |
| YCRGATCC | 8 | 238 | 4.90E-08 |
| GAAAGRAA | 8 | 334 | 7.30E-05 |
| TWCCATAT | 8 | 130 | 1.70E-04 |
| ACACAS | 6 | 968 | 2.10E-04 |
| CTGCAGY | 7 | 553 | 3.40E-04 |
| RGMAGAG | 7 | 1339 | 7.20E-04 |
| TGCRGGTT | 8 | 171 | 8.70E-04 |
| CWCATCT | 7 | 445 | 1.50E-03 |
| TTCCCAS | 7 | 659 | 1.40E-03 |
| TCATGGCA | 8 | 120 | 2.20E-03 |
| ACTAGTAT | 8 | 62 | 2.80E-03 |
| GSCGGCG | 7 | 158 | 3.60E-03 |
| GGAGCGGC | 8 | 66 | 3.90E-03 |
| AAAATGW | 7 | 801 | 4.40E-03 |
| YCYTCTCC | 8 | 420 | 4.90E-03 |
| CCCAGCSC | 8 | 205 | 5.60E-03 |
| CGTRTGT | 7 | 73 | 4.20E-03 |
| TTYDTTTC | 8 | 544 | 3.20E-05 |
| TATAGYTG | 8 | 108 | 8.60E-03 |
| CAGAGAKG | 8 | 140 | 1.20E-02 |
| GAGGDCAG | 8 | 232 | 2.00E-03 |
| GVAGGAGG | 8 | 307 | 8.80E-03 |
| CCCGYCTC | 8 | 79 | 1.30E-02 |
| CACCGSCC | 8 | 98 | 1.80E-02 |
| YTTCTGT | 7 | 617 | 2.10E-02 |
| GCRGGTTA | 8 | 66 | 2.10E-02 |
| CACRG | 5 | 2827 | 2.80E-02 |
| CGSAGGCG | 8 | 60 | 3.40E-02 |
| GAATGRAT | 8 | 193 | 4.80E-02 |
| Number of total motifs |  | 67123 |  |
| Number of motifs per promoter |  | 51 |  |
