# Supplementary material for: Characterization of gene promoters in pig: conservative elements, regulatory motifs and evolutionary trend
Source: PeerJ. 2019 Jun 25;7:e7204. doi: 10.7717/peerj.7204 (PMC6598670; doi:10.7717/peerj.7204)
Supplement: Supplemental Information 6 — aThe value gives the average and standard error of mean. bThe P-value was calculated based on the Mann-Whitney test. [file peerj-07-7204-s006.docx]

| Terms | HK genes | TS genes | *P-*value^b^ |
| --- | --- | --- | --- |
| dN | 0.084 ± 0.012^a^ | 0.27±0.031 | 3.54E-59 |
| dS | 0.82 ± 0.044 | 1.82 ± 0.226 | 1.84E-27 |
| dN/dS | 0.12 ± 0.011 | 0.20±0.024 | 1.34E-36 |
| dP | 0.43 ±0.022 | 0.64±0.041 | 2.72E-43 |
